# Misconceptions About E-cigarettes Among Healthcare Workers at Primary Health Care Centers in Riyadh, Saudi Arabia

**DOI:** 10.7759/cureus.77051

**Published:** 2025-01-06

**Authors:** Issa M Alkhonain, Mohammad Alqahtani, Lara A Alnamlah, Mostafa Kofi

**Affiliations:** 1 Family and Community Medicine, Prince Sultan Military Medical City, Riyadh, SAU

**Keywords:** attitude, awareness, e-cigarettes, hcws, phcs

## Abstract

Background:As e-cigarettes become increasingly popular, especially among youth, addressing the misinformation surrounding their use is crucial. This study aims to enhance healthcare workers' knowledge of the risks associated with e-cigarettes, ultimately improving patient education and public health outcomes.

Aims and objectives: To assess health workers' awareness of misconceptions surrounding e-cigarette use and the implications of this awareness on their personal use and professional practices

Methods: A cross-sectional study was conducted at the primary healthcare centers of Prince Sultan Military Medical City (PSMMC) in Riyadh, Saudi Arabia, to assess healthcare professionals' awareness of e-cigarette misconceptions. Utilizing a convenience sampling technique, the target sample size was 246 participants, and the actual participants were 208, ensuring robust representation. Eligible participants include PSMMC healthcare providers currently working in PHC settings, while those from other departments or institutions will be excluded. Data was collected through a structured online questionnaire covering demographic information, personal e-cigarette usage, awareness of health impacts, addictive potential, and regulations related to e-cigarettes.

Results: The study included a total of 208 participants, with the majority (150 participants, 72.1%) aged between 25 and 35 years. The group was predominantly male (108 participants, 51.9%) and Saudi nationals (147 participants, 70.7%). Additionally, 108 participants (51.9%) were married, and half were specializing in family medicine. While only 35 (16.8%) personally use e-cigarettes, 124 (59.6%) know someone who does, and social media is the primary information source for 127 (61.06%) participants. A strong majority (185,88.9%) believe e-cigarettes are harmful, and 164 (78.8%) recognize the potential harm to bystanders from e-cigarette aerosol. The views on e-cigarettes being less harmful than traditional cigarettes are mixed, with 64 (30.8%) in agreement, while 136 (65.4%) acknowledge their addictive nature. There is widespread support for regulating e-cigarettes like tobacco products (150, 72.1%) and prohibiting their use for individuals under 18 (164, 78.8%). Statistical analysis shows a significant association between the specialty of family medicine and the belief in the health risks of e-cigarettes (p=0.029), indicating the need for targeted education and policy measures.

Conclusion:Despite participants' relatively good knowledge of e-cigarettes and their health implications, a significant number of misconceptions persist. This highlights the need for ongoing education and training programs tailored for healthcare professionals to address gaps in understanding and ensure they are equipped with accurate information.

## Introduction

In recent years, the use of e-cigarettes has surged, particularly among youth, transforming from a tool for smoking cessation into the most commonly used tobacco product in this demographic [1]. Initially marketed as a safer alternative to traditional cigarettes, e-cigarettes were embraced by many as a means to quit smoking. However, a growing body of research has raised significant concerns regarding their safety and efficacy, particularly for young adults, pregnant individuals, and those who do not currently use tobacco products. The Centers for Disease Control and Prevention (CDC) stated in 2024 that e-cigarettes were unsafe for these groups, emphasizing that the benefits and harms associated with their use as a cessation method remain poorly understood, and the U.S. Food and Drug Administration (FDA) has yet to approve e-cigarettes for this purpose [1].

The literature surrounding e-cigarettes has expanded rapidly, revealing a concerning trend: the negative effects of e-cigarette use often outweigh any potential benefits. A review conducted by Coral in 2022 highlighted that existing evidence does not support the effectiveness of e-cigarettes as a strategy for aiding in the cessation of traditional tobacco smoking. This is particularly alarming given the emerging adverse effects on lung health associated with e-cigarette use [2]. Numerous studies have identified serious complications linked to e-cigarettes, including E-cigarette or vaping product use-associated lung injury (EVALI), nicotine toxicity, burns, and injuries related to e-cigarette batteries [3].

The emergence of EVALI as a recognized health issue underscores the dangers associated with vaping. In 2019, the Wisconsin and Illinois Departments of Public Health reported 98 cases of lung injuries of unknown origin, which were potentially associated with vaping products. This led to the identification of EVALI as a new clinical entity [4]. Additionally, a systematic review conducted by Seitz et al. in 2018 examined burn injuries caused by e-cigarette explosions [5]. Among 164 cases analyzed, a significant proportion (65%) of e-cigarettes exploded while in pockets, with the majority of injuries classified as second-degree burns (35%) or a combination of second and third-degree burns (20%) [5].

Beyond physical health risks, the implications of e-cigarette use extend to mental health and vascular health. A review by Javed et al. in 2022 found that adolescents who use e-cigarettes are at a higher risk of experiencing mental health issues, including depression and suicidality [6]. Similarly, Pincus et al. review of 40 publications indicated that e-cigarette use induces oxidative stress, leading to endothelial damage and potentially resulting in erectile dysfunction [7]. Furthermore, a study by Brett et al. investigating clinicians' perspectives on e-cigarettes as a smoking cessation tool for cancer patients revealed a consensus against recommending e-cigarettes for this purpose [8].

Despite the mounting evidence of harm associated with e-cigarette use, misconceptions persist, particularly among young adults. A study by Alzahrani et al. in 2021 assessed the knowledge of medical students regarding e-cigarettes and found several misconceptions and a lack of awareness about their harmful effects [9]. Similarly, a cross-sectional study conducted by Almutham et al. in 2018 among medical students at Alqasim University revealed a significant gap in knowledge about e-cigarettes [10]. This lack of understanding extends to health professionals, as demonstrated by Alsanea et al. across five different health colleges, which found that insufficient knowledge and misconceptions about e-cigarettes negatively influenced students' attitudes toward their use, potentially leading to adverse public health outcomes [11]. Additionally, a 2017 study highlighted a knowledge gap among physicians and pharmacists regarding e-cigarettes, which adversely affected their ability to counsel patients effectively [12]. A more recent study conducted in Turkey in 2022 revealed a lack of confidence among family physicians when counseling patients about smoking cessation strategies, further emphasizing the need for improved education on e-cigarettes [13]. The perception also has been studied in a paper that was conducted among chest health care providers about the use of E-cig and confirmed that there is variability in the perception and beliefs; however, there were many of those participants recommending against the use of these techniques as harmful reduction of tobacco smoke [14]. A study made in the USA clarified that there is no association between E-cig use and smoking cessation [15].

As e-cigarettes gain popularity, especially among youths, it is crucial to address the misinformation that perpetuates their use. Given the evolving nature of tobacco products, healthcare providers must remain informed and equipped to offer guidance. This study seeks to bridge gaps in knowledge among healthcare workers regarding the risks associated with e-cigarette use, leading to improved patient education and public health outcomes. Additionally, the results will underscore the necessity for targeted educational initiatives to combat misconceptions and promote informed decision-making.

Ultimately, the findings of this study could serve as a significant step toward improving public health initiatives related to tobacco control and encouraging a more health-conscious approach among both healthcare providers and the public at large.

The primary objective of the study is to assess health workers' awareness of misconceptions surrounding e-cigarette use and the implications of this awareness on their personal use and professional practices. Furthermore, the study will aim to evaluate the need for educational programs focused on e-cigarette misconceptions.

## Materials and methods

Methodology

A cross-sectional study was conducted at the PHC centers of Prince Sultan Military Medical City (PSMMC) in Riyadh, Kingdom of Saudi Arabia (KSA). Cross-sectional studies are advantageous due to their ability to provide a snapshot of the participants' awareness at a single point in time, allowing for the examination of patterns and associations. The data collection was started on the 1st of July 2024 and continued until the 1st of October 2024, with a total of 208 participants. Prince Sultan Military Medical City Scientific Research Center approved the study with an approval number E-2323.

Sample and sampling technique

The sample consisted of healthcare professionals working in PHC at PSMMC. We employed a convenience sampling technique to enroll participants, we choose this method as it allows for flexible recruitment based on availability. The target sample size is 246, calculated based on the total number of healthcare providers at PHCs with an existing literature on prevalence and proportions by using one proportion formula, ensuring a robust representation of the population.

Inclusion and exclusion criteria

Inclusion criteria will define eligible participants as healthcare providers, including (physicians, nurses, pharmacists, radiology technicians, and lab technicians) who are currently working within the PHC settings at Prince Sultan Medical Military City. Any employee at PHCs outside healthcare fields, such as (IT, secretaries, housekeeping, etc.) will be excluded from the study.

Data collection method

Data was collected through a structured online questionnaire formatted for easy response via e-mail or WhatsApp. The questionnaire, adapted from Wojciech S. Zgliczyński’s study on physicians' knowledge and beliefs about e-cigarettes in Poland, was validated in the same study with Kappa coefficients for critical questions ranging from 0.88 to 0.96 [16]. It includes the following sections.

Demographic Data

This section gathered essential demographic information, including age, sex, nationality, marital status, specialty, position, and years of experience in healthcare.

E-Cigarette Usage

Respondents were asked about their personal use of e-cigarettes, frequency of use, and the influence of peers on their behavior.

Awareness and Knowledge

This critical section assessed the participants' awareness of the health impacts of e-cigarettes, their addictive potential, and current regulations. Questions encompass common misconceptions and factual statements to evaluate knowledge accurately.

Educational Needs

Lastly, participants were inquired about their perceptions of their educational needs regarding e-cigarettes and their willingness to participate in potential training programs.

Data analysis

Once collected, responses will be analyzed using SPSS version 21. Descriptive statistics will provide insight into demographic trends, while inferential statistics will help determine associations between awareness levels and e-cigarette usage among healthcare providers, and the chi-square test will be used to determine the p-value and consider the significance if less than 0.05.

## Results

In Table 1, demographic data reveals a young workforce, with 72.1% of participants aged 25-35, primarily comprising males (51.9%) and Saudis (70.7%). A notable 51.9% are married, and half specialize in family medicine. The majority have 1-10 years of experience (76%).

**Table 1:** Sociodemographic characteristics of participants (n=208).

Parameters		Number	Percentage
Age	25 – 35	150	72.1
	36 - 45	41	19.7
	46 - 55	10	4.8
	56 - 60	7	3.4
Gender	Male	108	51.9
	Female	100	48.1
Nationality	Saudi	147	70.7
	Non-Saudi	61	29.3
Marital status	Married	108	51.9
	Single	95	45.7
	Divorced	5	2.4
Specialty	Family medicine	105	50.5
	nurse	78	37.5
	Pharmacist	25	12.0
Position	Charge nurse	7	3.4
	Consultant	19	9.1
	Pharmacist	12	5.8
	Registrar	66	31.7
	Senior registrar	19	9.1
	Staff nurse	73	35.1
	Tech	12	5.8
Years of experience	1 _ 10	158	76.0
	11 20	38	18.3
	More than 20	12	5.8

**Figure 1:**
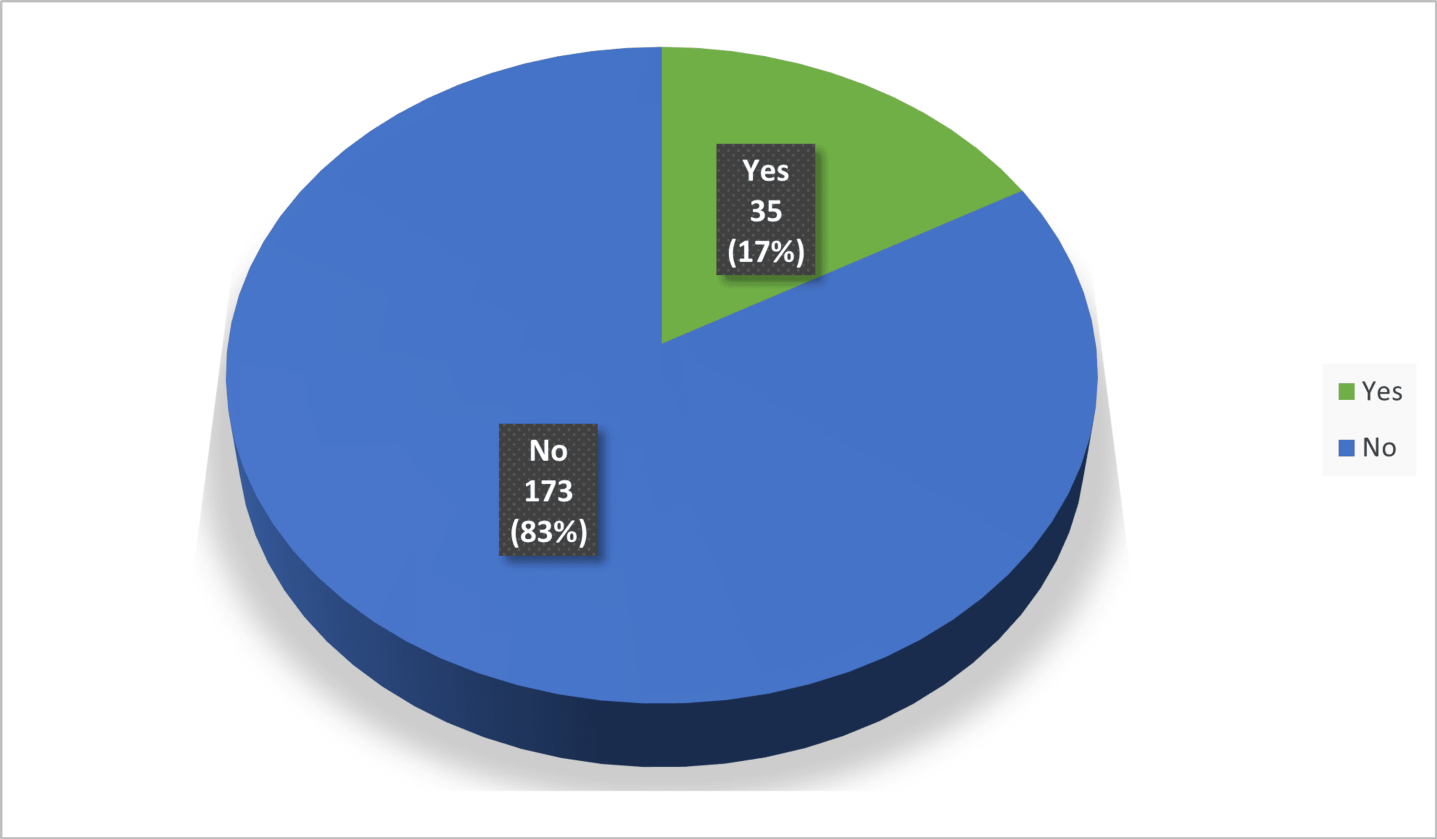
Use of E-Cigarettes among participants (n=208)

**Figure 2:**
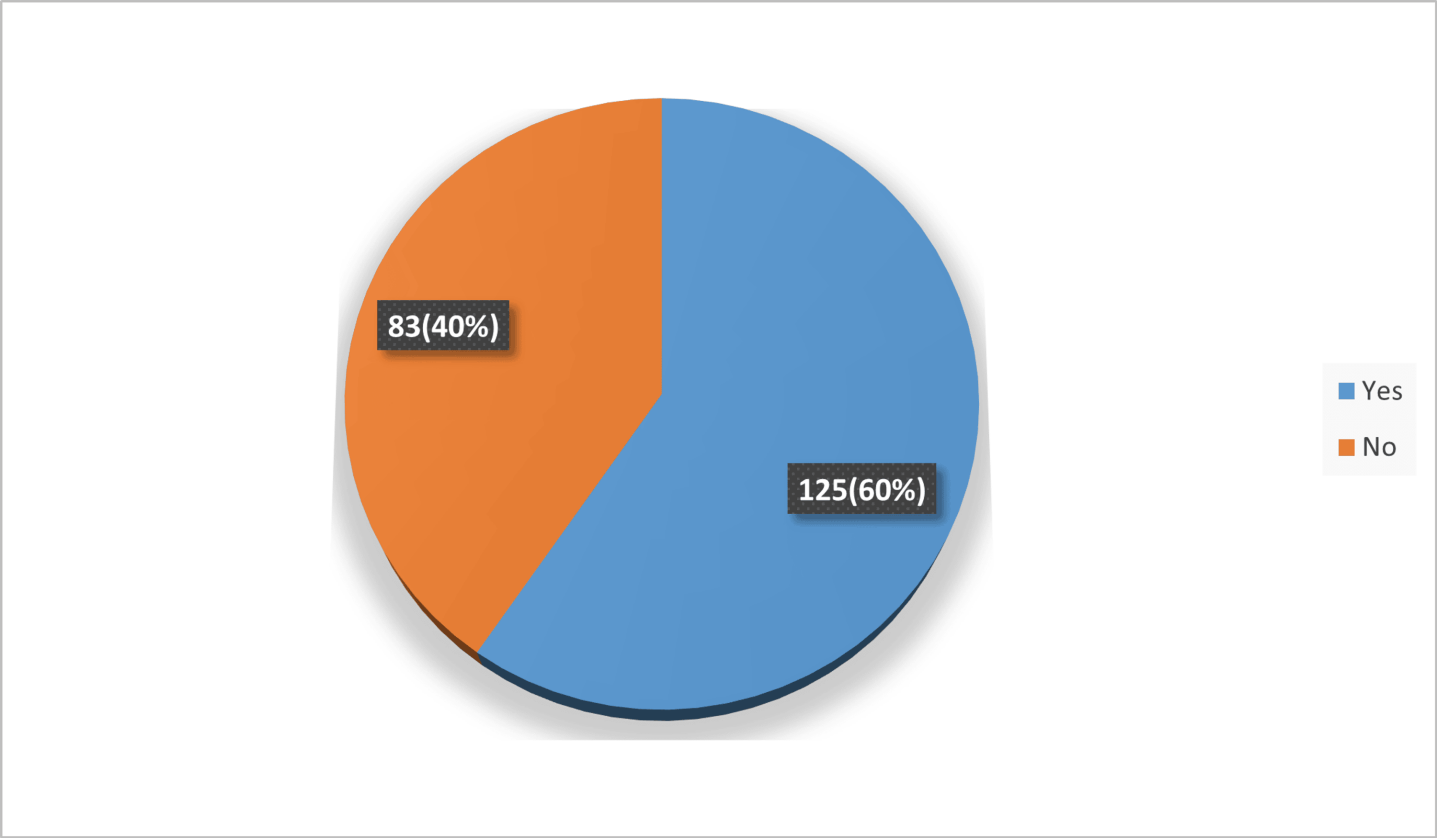
Participants know someone who use e-cigarettes (n=208).

**Figure 3:**
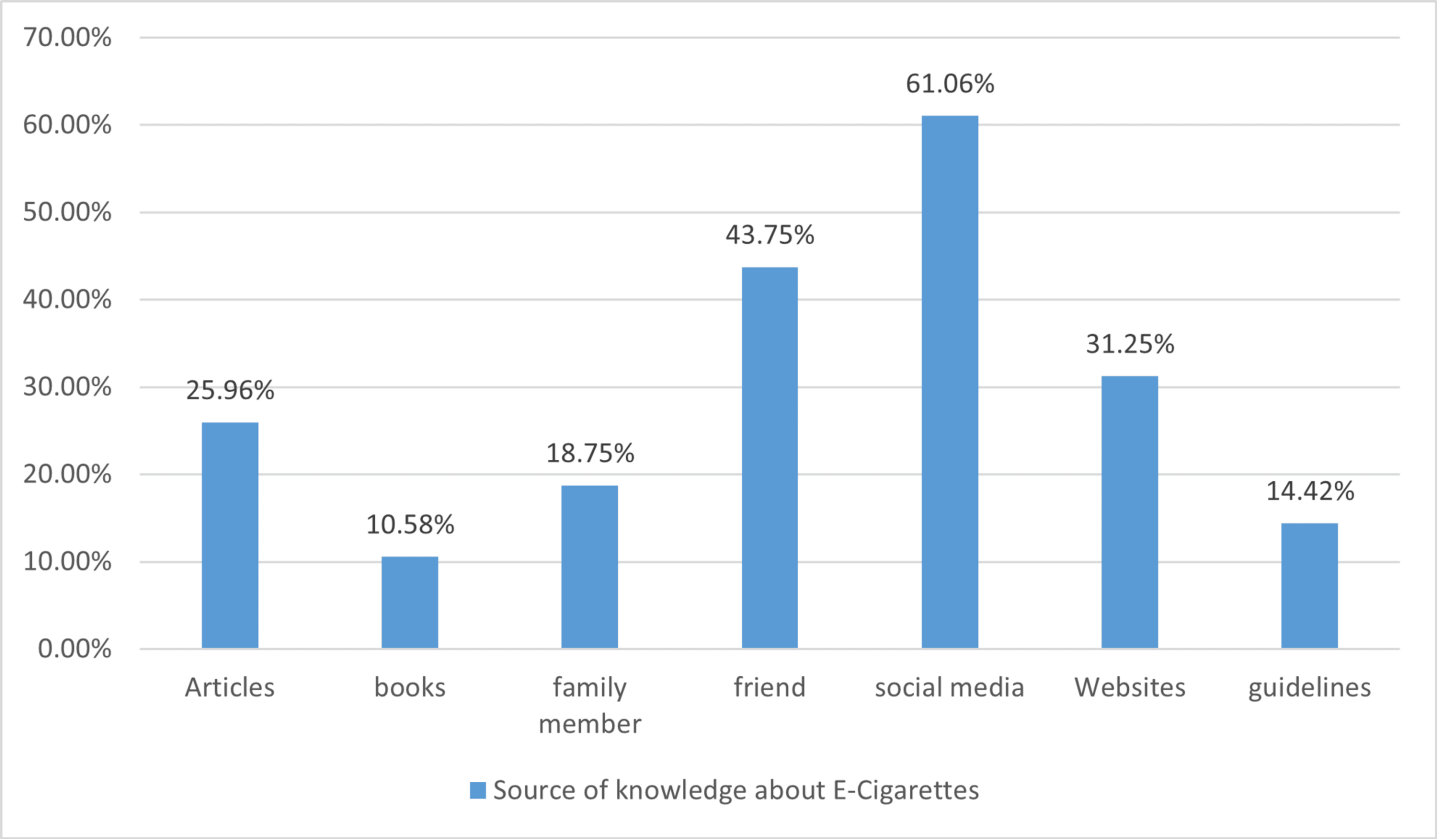
Participants’ source of knowledge about e-cigarettes (n=208).

In Figures 1-3, only 16.8% of respondents personally use e-cigarettes, while a significant 59.6% know someone who does. Social media stands out as the primary source of information about e-cigarettes, cited by 61.06% of participants. Meanwhile, traditional sources like books and articles play a lesser role, highlighting a shift towards digital platforms for health information.

Table 2 shows that a substantial 88.9% of respondents firmly believe that e-cigarette use is harmful to the health of the use. Furthermore, 78.8% of participants recognize that e-cigarette aerosol can harm bystanders. Interestingly, the debate about whether e-cigarettes are less harmful than traditional cigarettes remains divided. While 30.8% of respondents agree that e-cigarettes pose a lower risk, a significant portion of the population holds contrary views. Additionally, the perception of e-cigarettes as a gateway to conventional cigarette use and their addictive nature cannot be overlooked, with 65.4% acknowledging the addictive potential of e-cigarettes. There is a unified sentiment regarding the need for regulation: 72.1% support treating e-cigarettes similarly to tobacco products. This indicates a consensus on the importance of implementing robust policies to mitigate associated health risks, particularly concerning minors, with 78.8% advocating for prohibiting e-cigarettes for individuals under 18.

**Table 2:** Knowledge of participants of online nutritional applications and tele-dietetics (n=208).

	Totally agree	Rather agree	Rather disagree	Totally disagree
E-cigarette use is harmful to health of the user	185 (88.9%)	16 (7.7%)	6 (2.9%)	1 (0.5%)
E-cigarette aerosol is harmful to people in the vicinity of the users	164 (78.8%)	27 (13.0%)	17 (8.2%)	0 (0%)
E-cigarettes are less harmful than conventional cigarettes	64 (30.8%)	53 (25.5%)	45 (21.6%)	46 (22.1%)
E-cigarettes are carcinogenic	148 (71.2%)	44 (21.2%)	13 (6.3%)	3 (1.4%)
The risk of cancer is lower for the e-cigarettes than for the conventional cigarettes	58 (27.9%)	55 (26.4%)	60 (28.8%)	35 (16.8%)
E-cigarette use increases the risk of cardiovascular diseases, including myocardial infarction and stroke	155 (74.5%)	40 (19.2%)	12 (5.8%)	1 (0.5%)
The risk of cardiovascular diseases is lower for e-cigarettes than for conventional cigarettes	62 (29.8%)	55 (26.4%)	60 (28.8%)	31 (14.9%)
E-cigarette use increases the risk of chronic lung diseases, including COPD	159 (76.4%)	29 (13.9%)	17 (8.2%)	3 (1.4%)
The risk of chronic lung diseases is lower for the e-cigarettes than for the conventional cigarettes	57 (27.4%)	47 (22.6%)	62 (29.8%)	42 (20.2%)
E-cigarettes could be a, gateway to conventional cigarette use in the future	88 (42.3%)	57 (27.4%)	43 (20.7%)	20 (9.6%)
You can become addicted to the e-cigarette	136 (65.4%)	33 (15.9%)	25 (12.0%)	14 (6.7%)
E-cigarettes are less addictive than conventional cigarettes	50 (24.0%)	54 (26.0%)	62 (29.8%)	42 (20.2%)
E-cigarettes should be recommended as a smoking cessation tool	43 (20.7%)	34 (16.3%)	54 (26.0%)	77 (37.0%)
Smokers who do not want to quit smoking should be offered and encouraged to use e-cigarettes	41 (19.7%)	33 (15.9%)	66 (31.7%)	68 (32.7%)
Smokers who failed to quit with conventional smoking cessation should be offered and encouraged to use e-cigarettes	33 (15.9%)	45 (21.6%)	64 (30.8%)	66 (31.7%)
I recommend the e-cigarettes to my patients smoking conventional cigarettes	39 (18.8%)	27 (13.0%)	52 (25.0%)	90 (43.3%)
Discussing e-cigarettes with patients may encourage them to use e-cigarettes	48 (23.1%)	42 (20.2%)	61 (29.3%)	57 (27.4%)
E-cigarette promotion and advertising should be banned	127 (61.1%)	50 (24.0%)	20 (9.6%)	11 (5.3%)
E-cigarettes should be prohibited to minors (under 18 years)	164 (78.8%)	25 (12.0%)	13 (6.3%)	6 (2.9%)
E-cigarette use in public places should be banned	141 (67.8%)	38 (18.3%)	20 (9.6%)	9 (4.3%)
E-cigarette use should be banned indoors	140 (67.3%)	45 (21.6%)	16 (7.7%)	7 (3.4%)
E-cigarettes should be regulated in the same way as tobacco products	150 (72.1%)	34 (16.3%)	19 (9.1%)	5 (2.4%)

Table 3 shows the p-values indicating varying degrees of agreement with the statement that e-cigarettes are harmful to health, with a significant association observed in the specialty of family medicine (p=0.029). Most other categories show higher p-values, suggesting that demographic factors may not significantly influence opinions on e-cigarette harm. 

**Table 3:** Knowledge of the harmful effect of e-cigarettes in association with sociodemographic characters of participants’ (n=208). The Chi-square test was used. p-value is considered significant if less than 0.05.

Parameters		e-cigarettes are harmful				Total (n=208)	p-value
		Totally agree	Rather agree	Rather disagree	Totally disagree		
Age	25 - 35	133	133	1	5	150	0.725
		71.9%	71.9%	100.0%	83.3%	72.1%	
	36 - 45	36	36	0	0	41	
		19.5%	19.5%	0.0%	0.0%	19.7%	
	46 - 55	9	9	0	1	10	
		4.9%	4.9%	0.0%	16.7%	4.8%	
	56 - 60	7	7	0	0	7	
		3.8%	3.8%	0.0%	0.0%	3.4%	
Gender	Male	10	10	5	0	108	0.224
		62.5%	62.5%	83.3%	0.0%	51.9%	
	Female	6	6	1	1	100	
		37.5%	37.5%	16.7%	100.0%	48.1%	
Nationality	Saudi	13	13	6	0	147	0.114
		81.3%	81.3%	100.0%	0.0%	70.7%	
	Non-Saudi	3	3	0	1	61	
		18.8%	18.8%	0.0%	100.0%	29.3%	
Marital status	Married	9	9	3	1	108	0.873
		56.3%	56.3%	50.0%	100.0%	51.9%	
	Single	6	6	3	0	95	
		37.5%	37.5%	50.0%	0.0%	45.7%	
	Divorced	1	1	0	0	5	
		6.3%	6.3%	0.0%	0.0%	2.4%	
Specialty	Family medicine	4	4	3	0	105	0.029
		25.0%	25.0%	50.0%	0.0%	50.5%	
	Nurse	6	6	3	1	78	
		37.5%	37.5%	50.0%	100.0%	37.5%	
	Pharmacist	6	6	0	0	25	
		37.5%	37.5%	0.0%	0.0%	12.0%	
Years of nursing experience	1 _ 10	11	11	4	1	158	0.623
		68.8%	68.8%	66.7%	100.0%	76.0%	
	11 20	5	5	2	0	38	
		31.3%	31.3%	33.3%	0.0%	18.3%	
	More than 20	0	0	0	0	12	
		0.0%	0.0%	0.0%	0.0%	5.8%	
Position	Charge nurse	1	1	0	0	7	0.318
		6.3%	6.3%	0.0%	0.0%	3.4%	
	Consultant	0	0	1	0	19	
		0.0%	0.0%	16.7%	0.0%	9.1%	
	Pharmacist	4	4	0	0	12	
		25.0%	25.0%	0.0%	0.0%	5.8%	
	Registrar	4	4	2	0	66	
		25.0%	25.0%	33.3%	0.0%	31.7%	
	Senior registrar	0	0	0	0	19	
		0.0%	0.0%	0.0%	0.0%	9.1%	
	Staff nurse	5	5	3	1	73	
		31.3%	31.3%	50.0%	100.0%	35.1%	
	Tech	2	2	0	0	12	
		12.5%	12.5%	0.0%	0.0%	5.8%	

Table 4 shows a significant p-value of 0.029 for gender, suggesting males tend to agree more than females. Similarly, the p-value of 0.037 for nationality indicates a higher level of agreement among Saudis compared to non-Saudis. Other factors, such as age and marital status, showed no significant associations, with p-values of 0.709 and 0.856, respectively.

**Table 4:** Knowledge of the carcinogenic effect of e-cigarettes in association with sociodemographic characters of participants’ (n=208). The Chi-square test was used. p-value is considered significant if less than 0.05.

Parameters		e-cigarettes are carcinogenic				Total (n=208)	p-value
		Totally agree	Rather agree	Rather disagree	Totally disagree		
Age	25 - 35	103	32	13	2	150	0.709
		69.6%	72.7%	100.0%	66.7%	72.1%	
	36 - 45	31	9	0	1	41	
		20.9%	20.5%	0.0%	33.3%	19.7%	
	46 - 55	8	2	0	0	10	
		5.4%	4.5%	0.0%	0.0%	4.8%	
	56 - 60	6	1	0	0	7	
		4.1%	2.3%	0.0%	0.0%	3.4%	
Gender	Male	74	23	11	0	108	0.029
		50.0%	52.3%	84.6%	0.0%	51.9%	
	Female	74	21	2	3	100	
		50.0%	47.7%	15.4%	100.0%	48.1%	
Nationality	Saudi	100	33	13	1	147	0.037
		67.6%	75.0%	100.0%	33.3%	70.7%	
	Non-Saudi	48	11	0	2	61	
		32.4%	25.0%	0.0%	66.7%	29.3%	
Marital status	Married	77	23	7	1	108	0.856
		52.0%	52.3%	53.8%	33.3%	51.9%	
	Single	66	21	6	2	95	
		44.6%	47.7%	46.2%	66.7%	45.7%	
	Divorced	5	0	0	0	5	
		3.4%	0.0%	0.0%	0.0%	2.4%	
Specialty	Family medicine	74	24	6	1	105	0.792
		50.0%	54.5%	46.2%	33.3%	50.5%	
	Nurse	58	13	5	2	78	
		39.2%	29.5%	38.5%	66.7%	37.5%	
	Pharmacist	16	7	2	0	25	
		10.8%	15.9%	15.4%	0.0%	12.0%	
Years of nursing experience	1 _ 10	113	33	10	2	158	0.949
		76.4%	75.0%	76.9%	66.7%	76.0%	
	11 20	26	8	3	1	38	
		17.6%	18.2%	23.1%	33.3%	18.3%	
	more than 20	9	3	0	0	12	
		6.1%	6.8%	0.0%	0.0%	5.8%	
Position	Charge nurse	4	2	1	0	7	0.677
		2.7%	4.5%	7.7%	0.0%	3.4%	
	Consultant	15	2	2	0	19	
		10.1%	4.5%	15.4%	0.0%	9.1%	
	Pharmacist	9	2	1	0	12	
		6.1%	4.5%	7.7%	0.0%	5.8%	
	Registrar	44	18	4	0	66	
		29.7%	40.9%	30.8%	0.0%	31.7%	
	Senior registrar	15	3	0	1	19	
		10.1%	6.8%	0.0%	33.3%	9.1%	
	Staff nurse	54	12	5	2	73	
		36.5%	27.3%	38.5%	66.7%	35.1%	
	Tech	7	5	0	0	12	
		4.7%	11.4%	0.0%	0.0%	5.8%	

## Discussion

In our study, we observed that only 16.8% of participants reported personal use of e-cigarettes, contrasting sharply with the fact that a notable 59.6% are acquainted with individuals who do engage in this practice. This disparity underscores the pervasive nature of e-cigarette usage within communities, even among those who do not partake themselves. Furthermore, participants demonstrated a pronounced awareness of the potential health risks associated with e-cigarette use; an impressive 88.9% expressed firm beliefs that e-cigarettes are detrimental to the health of users. This conviction extends beyond users themselves, as 78.8% of respondents acknowledged the potential harm that e-cigarette aerosol poses to bystanders.

Such a high level of awareness and concern about the health implications of e-cigarettes reflects a significant understanding of tobacco-related issues among the surveyed population, particularly among health professions students. Previous studies have similarly shown that this demographic possesses substantial knowledge regarding e-cigarettes. For instance, over 70% of participants in our research were cognizant that e-cigarettes contain nicotine, are classified as tobacco products, and carry oncogenic risks [17]. This is in stark contrast to findings from Hangzhou University in China, where the comprehension of e-cigarettes among students was markedly lower, with only approximately 58% acknowledging that e-cigarettes contain nicotine and more than 68% failing to recognize them as tobacco products [18].

While 30.8% of respondents acknowledge that e-cigarettes may pose a lower risk than traditional cigarettes, a substantial portion of the population possesses reservations. This dichotomy in perceptions highlights the ongoing debate surrounding the safety and implications of e-cigarette use, particularly given the societal concerns about their potential role as a gateway to conventional cigarette smoking. Notably, 65.4% of respondents recognized the addictive nature of e-cigarettes, reflecting a growing acknowledgment of their risks. Despite this awareness, approximately 48.5% of participants reported being unaware of the addictive properties of e-cigarettes. This knowledge gap is particularly alarming, as the addictiveness of nicotine in these products is a major health concern, especially among youth. The potential for lifelong dependence is accentuated, given that studies have demonstrated that serum nicotine levels associated with e-cigarette use can be remarkably similar to those found in traditional cigarette consumption [19]. This equivalence reinforces the notion that e-cigarettes are not a benign alternative to smoking but may serve as a conduit to sustained nicotine addiction.

Further complicating the narrative around e-cigarettes is the widespread uncertainty regarding their regulatory status. Our investigation revealed that nearly 90% of participants were unsure whether e-cigarettes had received approval from the U.S. Food and Drug Administration (FDA) as smoking cessation aids [17]. It is crucial to note that, as of the publication date of this study, no e-cigarette products have attained FDA approval for this purpose [20]. Such uncertainty parallels findings from a study conducted at a Saudi university, where a majority of medical students were similarly uninformed about the FDA’s stance on e-cigarettes as cessation aids [9].

Moreover, existing literature adds weight to the concerns regarding e-cigarette use as a smoking cessation strategy. A comprehensive meta-analysis encompassing 26 studies has indicated that adult smokers who utilize e-cigarettes for the purpose of quitting smoking are less likely to successfully cease smoking compared to their non-e-cigarette-using counterparts [19]. This evidence suggests that reliance on e-cigarettes may not be an effective or safe approach to smoking cessation, further underscoring the need for better education and more rigorous regulatory oversight in the realm of nicotine delivery systems.

The increasing prevalence of e-cigarettes among young adults has sparked significant concern regarding their perceived safety compared to conventional cigarettes. In a previous study, a majority of students expressed the belief that e-cigarettes are a safer alternative to traditional tobacco products [17]. This perception is echoed in a similar investigation conducted among university students in Jordan, where a significant number of participants also regarded e-cigarette use as less hazardous than smoking conventional cigarettes [21]. However, this belief is misleading; emerging evidence indicates that e-cigarette vapor contains harmful substances that can lead to severe health issues, including oral squamous cell carcinoma [22].

Furthermore, the nicotine present in e-cigarettes is not only highly addictive but has also been implicated in the development of oral cancers [23]. The neurobiological effects of nicotine are particularly concerning, especially for adolescents and young adults, whose brains continue to mature until approximately the age of 25. Research has demonstrated that nicotine exposure during this critical developmental period can disrupt the functioning of brain regions associated with impulse control, attention, mood regulation, and learning, thereby increasing the risk of future substance use disorders [24].

Moreover, the association between e-cigarette use and the onset of eating disorders, such as anorexia nervosa, binge eating, and bulimia, has been documented in studies involving college students in the United States [25]. These findings underscore the urgent need for regulatory measures and educational initiatives aimed at mitigating the risks associated with e-cigarette consumption.

In our study, we identified social media as the predominant source of information about e-cigarettes, with 61.06% of participants citing it as their primary reference. This trend signifies a notable shift from traditional informational sources, such as books and academic articles, towards digital platforms for health-related information. Supporting this observation, an online survey of e-cigarette users revealed that 35% learned about e-cigarettes from personal contacts, while 41% obtained information from the internet, and only a small percentage (10%) relied on other media sources, with 8% witnessing e-cigarette use firsthand [26].

Interestingly, a study conducted in Minnesota found that nearly all healthcare providers (92%) were aware of e-cigarettes. Yet, the primary sources of their information included patients, news reports, advertisements, and the internet rather than professional or academic literature [27]. This reliance on non-professional sources highlights a critical gap in the dissemination of accurate, evidence-based information about e-cigarettes among both the public and healthcare providers [28].

In our study, the analysis of p-values revealed varying levels of consensus regarding the perception that e-cigarettes are detrimental to health. Notably, a significant correlation was established within the family medicine specialty (p=0.029). Conversely, other categories demonstrated elevated p-values, suggesting that demographic variables may exert a minimal influence on attitudes toward the health risks associated with e-cigarette use. Previous research has indicated that opinions regarding e-cigarettes can vary based on age, marital status, and smoking habits. Specifically, younger individuals, married persons, and smokers tend to exhibit distinct perspectives on e-cigarettes, likely shaped by their unique experiences and personal circumstances [29]. Moreover, studies have highlighted that e-cigarette use is notably prevalent among adolescents, attributed to the devices' accessibility, their perceived safety compared to traditional cigarettes, and their appealing design [30]. Additional determinants, such as peer influence and curiosity, play a significant role in the decision to initiate e-cigarette use [31].

In the context of adult smokers grappling with substance use disorders, it was found that those who concurrently used e-cigarettes and traditional cigarettes demonstrated a higher likelihood of attempting to quit smoking in the past year. Furthermore, many of these individuals expressed a preference for e-cigarettes over conventional cessation aids, such as nicotine patches or gum, during their attempts to quit [32].

A previous study conducted in Saudi Arabia revealed a positive association between higher monthly income (5,001 SAR or more) and greater knowledge regarding e-cigarette use [29]. Interestingly, this study also found that individuals with higher incomes were more likely to engage in e-cigarette use [33]. This observation suggests that individuals belonging to higher socioeconomic strata may have enhanced access to educational resources or may exhibit greater receptivity to health information [34]. Such findings raise critical concerns regarding potential disparities in health literacy across various socioeconomic groups, underscoring the urgent need for targeted health education initiatives to address these gaps.

The implementation of targeted public health campaigns is essential for raising awareness about e-cigarette use in Saudi Arabia, particularly among youth and non-smokers. These initiatives should emphasize the health risks associated with e-cigarettes and challenge the misconception that they represent a safer alternative to traditional tobacco products. Additionally, comprehensive qualitative research is warranted to delve deeper into the factors that shape negative attitudes toward e-cigarette use, particularly within specific demographic segments.

Furthermore, regulating e-cigarette advertisements, especially those aimed at younger audiences, is crucial in mitigating the glamorization of these products. Initiatives must also focus on bridging the educational disparity regarding e-cigarette knowledge and usage that exists across different socioeconomic strata.

Healthcare providers play a pivotal role in this context. They should receive training on the potential utility of e-cigarettes in smoking cessation strategies, enabling them to offer tailored support to those seeking to quit. By implementing these recommendations, it is anticipated that e-cigarette usage will decline, thereby positively impacting smoking rates and health outcomes in the country. The medical education team, along with the department administration, will be involved in designing proper training for the healthcare providers.

Limitations 

Further studies need to be on a larger scale of sample size, including different departments and hospitals. In this study, we do not fully reach our target sample size; the literature needs more studies about e-cigarettes.

## Conclusions

Despite participants' relatively good knowledge of e-cigarettes and their health implications, a significant number of misconceptions persist. This highlights the need for ongoing education and training programs tailored for healthcare professionals to address gaps in understanding and ensure they are equipped with accurate information. Misconceptions can adversely affect health communication and patient care, making it crucial for healthcare workers to possess a nuanced understanding of e-cigarette risks and benefits. By fostering a more informed perspective, these professionals can better guide patients in making informed choices about tobacco and nicotine use. Additionally, addressing these misconceptions may enhance the overall public health response to vaping and its associated health concerns. Ultimately, this study underscores the importance of continuous education and proactive measures to dispel misinformation within healthcare settings.
